# Shrinkage Properties and Their Relationship with Degradation of Proteins Linking the Endomysium and Myofibril in Lamb Meat Submitted to Heating or Air Drying

**DOI:** 10.3390/foods11152242

**Published:** 2022-07-27

**Authors:** Weili Rao, Zhenxiao Shi, Sijia Liu, Ying Shu, Xiaoyu Chai, Zhisheng Zhang

**Affiliations:** College of Food Science and Technology, Hebei Agricultural University, Lekai South Avenue, Baoding 071000, China; iamraoweili@126.com (W.R.); a13231740168@126.com (Z.S.); liusijia131@126.com (S.L.); 15930431516@139.com (Y.S.); 15027708107@139.com (X.C.)

**Keywords:** shrinkage, lamb meat, endomysium, protein degradation, water bath heating, air drying

## Abstract

The shrinkage of the connective tissue and myofiber of lamb meat submitted to heat treatment or air drying at different storage stages (1, 5 and 7 days) was evaluated herein. The longitudinal and transverse shrinkage of heated lamb meat was significantly influenced by storage time and water bath heating temperature (50 °C, 70 °C and 90 °C) (*p* < 0.001). In contrast, the shrinkage of air-dried lamb meat was not influenced by storage time (*p* > 0.05). The microstructure of heated lamb meat, namely, the distance between muscle fascicles, the distance between myofibril networks, the area of myofibril networks, and the endomysium circumference, was significantly influenced by storage time (*p* < 0.05). During storage, the proportion of muscle fibers completely detached from endomysium increased, which could be due to the progressive degradation of proteins linking the endomysium and myofibril, including *β*-dystroglycan, *α*-dystroglycan, integrin-*β*1, and dystrophin. However, degradation of such proteins did not influence the shrinkage of lamb meat stored for five days or longer, since the decreased distance between myofibril networks indicated a higher shrinkage ratio of the endomysium compared to myofibers in samples air-dried at 35 °C or heated at 90 °C. The effect of these proteins on the shrinkage of heated lamb meat (raw meat stored for 1 day or less time) requires further elucidation.

## 1. Introduction

Muscle shrinkage during meat processing affects the energy consumption of meat products. For jerked beef, cured meat, air-dried meat, and other meat products that require desiccation, hot-air drying is commonly used in the industry. Long drying time and high energy consumption are recurrent issues in the application of hot-air drying in the food industry [1,2,3]. During hot drying, muscle tissue shrinks, especially the connective tissue on the surface of the meat, thus reducing the pore diameter between muscle fibers and hindering the migration of water molecules in myofibrils to the environment, which results in a long drying time and high energy consumption [1,2,4,5,6]. The air temperature used in hot-air drying is usually between 50–90 °C; within this temperature range, connective tissue shrinkage is caused by both heat and dehydration.

Muscle shrinkage during meat processing affects the eating quality of meat products. Tenderness is one of the most important sensory attributes related to beef and other meat products’ consumer acceptance [7]. Shrinkage and thermal dissolution of connective tissue during heating are directly related to meat tenderness. When heated below 60 °C, connective tissue contributes greatly to meat hardness. However, when connective tissue is heated above 60 °C, myofibrils contribute more significantly to meat hardness due to the thermal dissolution of collagen in the endomysium and perimysium [8]. However, due to shrinkage of the endomysium at 60 °C, more muscle fibers are found per unit volume, thus leading to increased meat hardness. Therefore, understanding the mechanism underlying meat shrinkage during processing is crucial.

In lamb meat, shrinkage that occurs during heating or drying could be related to the following three reasons: (i) shrinkage of the muscle myofibril network itself [9,10,11]; (ii) shrinkage of the connective tissue itself [12,13,14,15,16,17,18,19]; and (iii) shrinkage of connective tissue due to the muscle fibers’ shrinkage through the connection between muscle fibers and endomysium. The first and second reasons have been suggested in previous studies, even though these require more study. In contrast, the impact of the third presumed reason on meat shrinkage during heating or other processing conditions has been poorly studied. Individual muscle fibers connect to the endomysium mainly through two pathways, i.e., the desmin–integrin–laminin–collagen and the dystrophin–dystroglycan–laminin–collagen pathways [20]. These proteins, in particular dystrophin (Dys), *β*-dystroglycan (*β*-DG), *α*-dystroglycan (*α*-DG), and integrin-*β*1, have been shown to be susceptible to degradation with prolonged postmortem time. Thus, whether the degradation of these proteins influences the shrinkage of heated or air-dried meat needs to be further elucidated. In addition, it was described that *β*-DG, *α*-DG, integrin, laminin and other linking proteins between muscle fibers and endomysium could be hydrolyzed by the matrix metalloproteinases (MMPs) [21,22,23,24,25]. Previous studies have shown that MMP-2 enzyme activity increases gradually during postmortem storage [26], indicating that MMPs may continue to affect the processing characteristics of meat by degrading the extracellular matrix after slaughter. However, the overall activity of MMPs and the activity of other MMPs besides MMP-2 during postmortem aging time have not been assessed. To answer the above question, in the present study, the effect of heating or air drying (35 °C, below the denaturation temperature of protein in meat) on the shrinkage of lamb meat was studied separately. As described in research, hematoxylin–eosin staining is a common and suitable method to view the muscle fibers and connective tissue (endomysium and perimysium) of meat [27,28,29]. With ImageJ software, the porosity, cross-section of muscle fibers, distance between muscle bundles, circumference of endomysium, and distance between the muscle fibers of meat can be measured. By measuring these data, the shrinkage of muscle fibers or endomysiums can be easily observed. Meanwhile, the degree of degradation of linking proteins connecting the endomysium and myofibril during storage was evaluated in this study. If the distance between muscle fibers increases, it means the shrinkage of myofibril network is more than that of connective tissue, and the shrinkage of the endomysium is not affected by these proteins connecting the endomysium and myofibril. In addition, immunohistochemistry was used to explore the distance between the membrane and endomysium and the distribution of these linking proteins in meat samples. The distribution of these link proteins on the cell membrane can explain whether the pores between the muscle fibers are within the muscle cell or between the membrane and endomysium. Based on this information, we can predict whether this linking-protein degradation is more important for muscle shrinkage.

## 2. Materials and Methods

### 2.1. Sample Collection

Six small-tailed Han sheep (six months of age, male) raised within the same feedlot were slaughtered following Halal procedures at a commercial slaughterhouse (Jinhongqingzhen Co. Ltd., Baoding, China). After the carcasses were chilled at 4 °C for 12 h, *quadriceps femoris* muscles were excised from both sides of the carcasses, and the muscles were frozen at −38 °C for 6 h and stored at −18 °C. Prior to experiments, the muscle tissue was thawed at −0.5 °C and sliced into 1 cm cubes, and then stored for 1, 5, and 7 days. All procedures described herein were undertaken following the guidelines of the Animal Care and the Ethics Committee for Animal Experiments of the College of Food Science and Technology, Hebei Agricultural University, China. 

### 2.2. Heating, Air Drying, and Calculation of Shrinkage Ratio of Lamb Meat

Thirty meat cubes from six sheep (five replicates per sheep) were heated at 50 °C in water for 20 min, and then placed at room temperature, and the length, width and height were measured with a vernier caliper. Then, these samples were placed at 70 °C in a water bath for 20 min, and the procedure described above was repeated. Finally, these meat cubes were placed at 90 °C in a water bath and heated for 20 min, and the procedure described initially was repeated. After this step, the volume of the same was calculated.

For the air-drying treatment, 30 meat cube samples from six animals were placed on a perforated board and then into a dryer (D/GDWJB-100L, Dianhe Experimental Instrument Co. Ltd., Shanghai, China). The air-drying temperature was 35 °C. Air drying was halted when the moisture content of meat samples reached 50% (as calculated by the ratio between the weight of water and dried meat). The length, width, and height of air-dried meat cube samples were determined using a vernier caliper, and meat sample volume was calculated. Longitudinal, transverse, and volume shrinkage ratios of the meat cube samples were calculated using the following formula:

Shrinkage ratio = [(L1 − L2)/L1] × 100%

where L1 is the length, width, or volume of the meat samples before heating or air-drying treatments; and L2 is the length, width, or volume of the meat cubes after heating or air-drying treatments.

### 2.3. Microstructure Measurement

After heating or air-drying treatments, all meat samples were immediately fixed in 10% formaldehyde solution for two weeks. 0.5 mm thick cross-sections of fixed meat samples were obtained and dehydrated in a series of ethanol solutions of increasing concentration (70%, 80%, 90%, 95%, and anhydrous ethanol). After incubation with xylene, meat samples were embedded in wax, and 4 μm-thickness sections were obtained. The meat samples were subsequently stained with hematoxylin and eosin dyes. Xylene, ethanol, hematoxylin and eosin were purchased from Sinopharm (National Pharmaceutical Group Co. Ltd., Beijing, China).

Ten areas in each meat sample were surveyed using a microscope (Eclipse Ci, Nikon, Japan). The distance between muscle bundles and muscle fibers, porosity of lamb meat, area of muscle fibers, and endomysium circumference of muscle were calculated using the ImageJ software (V.1.8.0, National Institutes of Health, Bethesda, MD, USA). The scale bar was set at 50 μm, and magnification was 40 times. The distance between muscle bundles or between muscle fibers was measured based on ten pairs of adjacent muscle bundles or fibers selected in each image, upon which lines were drawn to connect the edges of two bundles or fibers using the mentioned software, and the length of this line was subsequently determined. Each pair of cells was measured twenty times, and data were recorded. The area of muscle fibers was calculated based on the diameter of twenty clear fibers uniformly selected in each image. Porosity was determined based on images converted into an 8-bit format, and fibers were marked in red with the threshold option; then, twenty areas in the image were selected (each area about 1/4 the size of the original image) to calculate porosity based on the following formula: porosity = (regional pore area)/(total regional area) × 100%. Finally, endomysium circumference was calculated following these steps: twenty areas were circled along the endomysium edge, and the number of endomysium structures in these areas was determined. Subsequently, the average area of each endomysium was calculated, and the radius and the circumference were calculated using the formulae s = πR^2^ and c = 2πR, respectively, in which S is the area, R is the radius, and C is the perimeter.

### 2.4. Western Blotting

Raw-lamb-meat samples were stored for a different number of days and were ground into powders with liquid nitrogen and mixed with a lysis buffer (Beijing Solaibao Technology Co., LTD., Beijing, China). Protein content was quantified with the BCA protein assay (Thermo Fisher Scientific Inc., Waltham, MA, USA). Western blotting was performed as described by Omairi et al. [30] with some modifications. The extracted protein supernatant was mixed with 5× protein-loading buffer at a ratio of 4:1 (*v*/*v*), boiled in a water bath for 5 min, and then stored at −20 °C for later use. SDS-PAGE gel preparation was conducted according to the manufacturer’s instructions. The sample volume in each well was 50 μg of protein. Protein samples were slowly transferred to a concentrated gel under constant voltage (80 V); when the desired band was transferred to the separation boundary layer of the concentrated gel, a constant voltage of 120 V was applied to the gel until the band reached the bottom of the gel. After electrophoresis, the sealed glass plate was opened, and the gel was carefully removed and placed onto a membrane. Antibodies anti-*β*-DG antibody (ab62373), anti-integrin *β*1 antibody (ab179471), anti-dystrophin antibody (ab275391), and anti-*α*-DG antibody (ab151979) were purchased from Abcam (Cambridge, MA, USA). After membrane transfer, the PVDF membrane was taken out and soaked in the sealing solution for 2 h at room temperature; then, the PVDF membrane was washed twice with Tris-buffered saline tween solution (TBST) for 5 min each. After the last washing, the corresponding primary antibody was added to the membrane and incubated overnight at 4 °C. Then, the membrane was soaked in TBST and washed in a shaker for 5 min. Subsequently, the membrane was incubated with the secondary antibody at room temperature under shaking for 2 h. The signal was automatically exposed and captured in an ECL immunofluorescence system (Tanon-5200 Multi, Tanon Science and Technology Co., Ltd., Shanghai, China).

### 2.5. Activity of Matrix Metalloproteinases (MMPs)

The activity of MMPs was measured using a commercial MMP ELISA Kit (Jiangsu Enzyme Immunity Industry Co., Ltd., Shanghai, China). The detection principle of the indicated kit was based on the encapsulation of phosphorylated antibodies; the more phosphorylated antibodies are captured, the higher the activity of MMPs. After meat samples were stored on different days, approximately 1 g of tissue was obtained and mixed with 9 mL of PBS buffer (pH 7.2–7.4) and left to stand for 10 min, followed by centrifugation at 3000 rpm for 20 min. Subsequently, the supernatant was collected and stored following the manufacturer’s instructions for subsequent analysis.

### 2.6. Immunohistochemistry Analysis

After dewaxing, glass slides were placed in a preheated antigen repair buffer (100 mM Tris, 5% [*w*/*v*] urea, pH 9.5, 95 °C) for 10 min. Glass slides were washed with PBS buffer for three consecutive times, with 5 min between each wash. Glass slides were incubated with the diluted antibody (dissolved in PBS solution containing 1% BSA and 0.1% Tween 20) and incubated for 1 h at room temperature or overnight at 4 °C, then washed with PBS solution for three times, with 5 min between each time, followed by incubation at room temperature with the secondary antibody (dissolved in 1% BSA) for 1 h. The secondary antibody solution was eliminated, and glass slides were washed with PBS three consecutive times with 5 min between each wash. 

### 2.7. Statistical Analysis

Two-way ANOVA with fixed factors of storage time (1, 5, and 7 days) and heating temperature (50 °C, 70 °C, and 90 °C) was used to analyze the effect of storage time, heating temperature and storage time × heating temperature on the longitudinal, transverse or volume shrinkage ratio of lamb meat, and was carried out in SPSS v.22.0 (IBM Corporation Inc., Armonk, NY, USA). General linear mode and least significant difference (LSD) were used as post hoc tests for ANOVA, and significant differences were considered at *p* values < 0.05. 

In addition, one-way ANOVA was used to analyze the effect of storage time on the longitudinal, transverse and volume shrinkage of air-dried lamb meat; the effect of storage time on the proportion of muscle fibers completely detached from the endomysium; and the effect of storage time on the content of active MMPs. Significant differences were considered when *p* values < 0.05. 

When heating, the meat cubes were consecutively submitted to three heating treatments: first, 50 °C heated for 20 min; then, 70 °C heated for 20 min; and, finally, 90 °C heated for 20 min. Due to heat and mass transfer, the outside and inside of the heated meat could be regarded as the result of different heat treatments, so one of the fixed factors of two-way ANOVA was heating treatment. The same went for the data of air-dried meat. Two-way ANOVA with fixed factors of storage time (1, 5 and 7 days) and heating treatment were used to analyze the effect of storage time, heating treatment and storage time × heating treatment on the structure of lamb muscle submitted to heating. Two-way ANOVA with fixed factors of storage time (1, 5 and 7 days) and air-drying treatment were used to analyze the effect of storage time, air-drying treatment and storage time × air-drying treatment on the structure of lamb muscle submitted to air drying. Significant differences were considered when *p* values were <0.05. 

## 3. Results

### 3.1. Shrinkage of Lamb Meat after Heat Treatment at Different Temperatures

Longitudinal, transverse, and volume shrinkage ratios of lamb meat samples were not significantly influenced by the interaction between storage time and heating temperature (Table 1). At 50 °C or 90 °C, the longitudinal-shrinkage ratio gradually increased as storage time increased, but was not significant. At 70 °C, the longitudinal-shrinkage ratios of lamb meat samples stored for 5 or 7 days were significantly higher than those in meat samples stored for 1 day. The longitudinal-shrinkage ratio was extremely significantly influenced by heating temperature (*p* < 0.001); the higher the heating temperature, the higher the longitudinal-shrinkage ratio of lamb meat. Transverse-shrinkage ratio was significantly influenced by the storage time of raw lamb meat (*p* = 0.001) or heating temperature (*p* < 0.001); the shorter the storage time or the higher the heating temperature, the greater the transverse-shrinkage ratio of lamb meat samples. The volume shrinkage ratio was not significantly influenced by the storage time of raw meat samples (*p* = 0.967) nor by the interaction between storage time and heating temperature (*p* = 0.427), but was significantly increased by heating temperature (*p* < 0.001); the higher the temperature, the greater the volume shrinkage ratio considering all storage time.

### 3.2. Shrinkage of Lamb Meat after Air-Drying Treatment

Storage time did not influence the longitudinal (*p* = 0.387), transverse (*p* = 0.390), or volume (*p* = 0.553) shrinkage ratios of lamb meat samples (Table 2). However, a downward trend in longitudinal-shrinkage ratio was observed as storage time increased.

### 3.3. Changes in the Microstructure of Lamb Meat after Heat or Air-Drying Treatment

**Figure 1:**
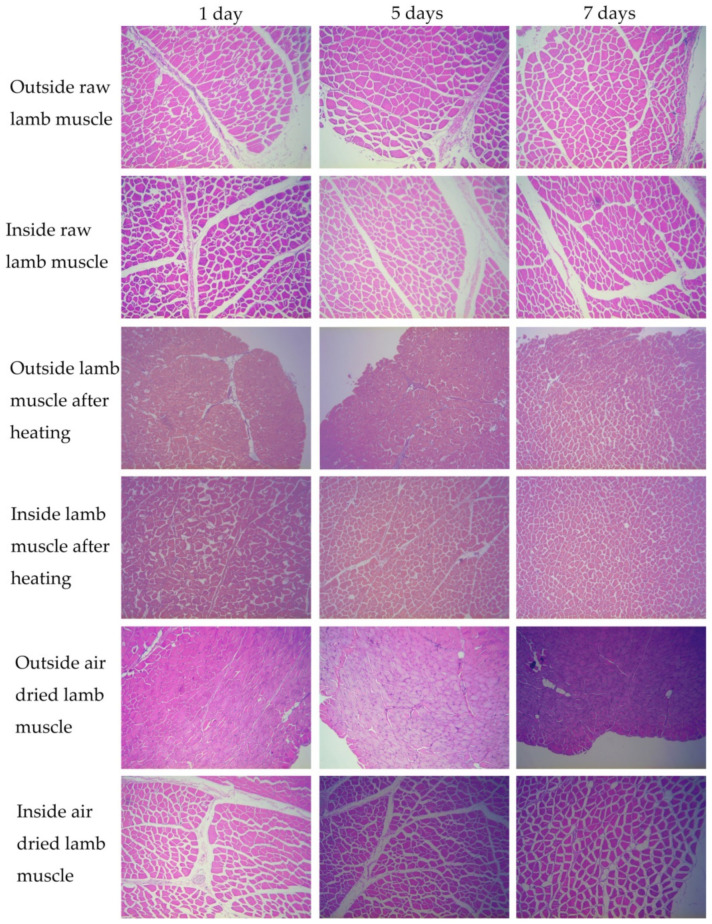
Effect of storage time (1, 5, and 7 days) on the microstructure of lamb muscle after water bath heating or air-drying treatment.

From histopathological observations, the gap between muscle bundles or between fibers was regular and visible in raw lamb meat on days 1, 5, or 7 of storage (Figure 1). Most muscle fibers were partly attached to endomysium structures, and only a few fibers were completely detached from the endomysium. Thus, storage time had a significant effect on the proportion of muscle fibers completely detached from the endomysium (*p* < 0.001); the longer the storage time, the greater the proportion of muscle fibers that completely detached from the endomysium (Table 3).

Similarly, the distance between muscle bundles (*p* = 0.002), the porosity of lamb meat (*p* < 0.001), the distance between myofibril networks (*p* < 0.001), the area of myofibril networks (*p* < 0.001), and the muscle’s endomysium circumference (*p* < 0.001) were significantly influenced by heat treatment of lamb meat (Table 4). The distance between muscle bundles (*p* < 0.001), the distance between myofibril networks (*p* = 0.001), the area of myofibril networks (*p* = 0.019), and the muscle’s endomysium circumference of muscle (*p* < 0.001) were significantly influenced by storage time, but not lamb meat porosity (*p* = 0.103). The interaction between storage time and heat treatment influenced the porosity of lamb meat (*p* = 0.005), but did not influence distance between muscle bundles (*p* = 0.424), distance between myofibril networks (*p* = 0.940), area of myofibril networks (*p* = 0.101), or muscle’s endomysium circumference (*p* < 0.449).

The distance between muscle bundles (*p* < 0.001), lamb meat porosity (*p* < 0.001), distance between myofibril networks (*p* < 0.001), area of myofibril networks (*p* < 0.001), and muscle’s endomysium circumference (*p* < 0.001) were significantly influenced by air drying (Table 4). The muscle’s endomysium circumference (*p* < 0.001) was significantly influenced by storage time, but the distance between muscle bundles (*p* = 0.411), distance between myofibril networks (*p* = 0.104), area of myofibril networks (*p* = 0.179), or the porosity of air-dried lamb meat (*p* = 0.058) were not influenced. The porosity of air-dried lamb meat (*p* = 0.008), area of myofibril networks (*p* < 0.001), and muscle endomysium circumference (*p* < 0.001) were influenced by the interaction between storage time and air drying; however, the distance between muscle bundles (*p* = 0.074) or the distance between myofibril networks (*p* = 0.474) were not influenced. In air-dried samples, the space between muscle bundles and between myofibril networks was reduced compared to raw lamb meat when storage time was 1 day, 5 days, or 7 days (Figure 1 and Table 4).

### 3.4. Degradation of Proteins Linking Endomysium and Myofibril during Storage

**Figure 2:**
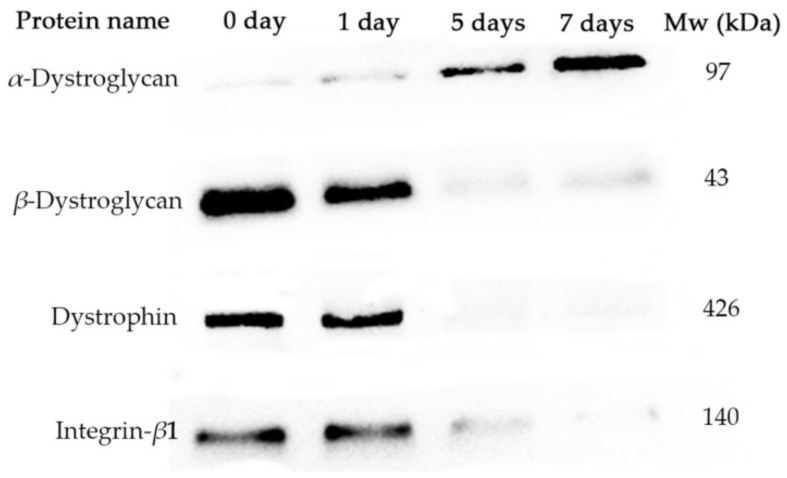
Effect of storage time (1, 5, and 7 day) on the degradation of proteins linking the endomysium and myofibrils of lamb meat.

Dystrophin and *β*-dystroglycan were impacted by storage time, and degradation of these protein bands was evident; the longer the storage time, the stronger the degradation of protein bands (Figure 2). Certain low-molecular-weight fragments resulting from protein degradation of *β*-DG (approximately 54, 39, and 35 kD) and dystrophin (approximately 260 kD or smaller) were detected. With increased storage time, the fluorescence intensity of protein bands of *α*-DG of 97 kDa gradually increased. 

*α*-DG is an extracellular peripheral glycoprotein [31]. In the present study, the anti-*α*-DG (ab151979) antibody against *α*-DG aa 471-752 was used to detect the 97-kDa protein without carbohydrate in its composition. With increased storage time, the content of protein composition of *α*-DG increased indicating degradation of carbohydrate composition.

**Figure 3:**
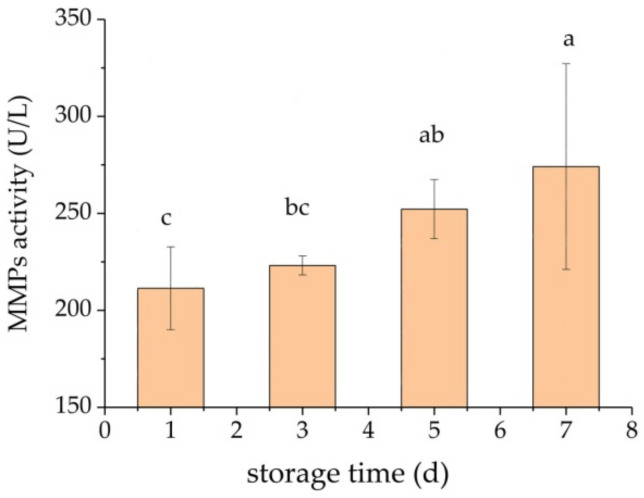
Effect of storage time (1, 5, or 7 days) on the activity of metalloproteinases (MMPs) in lamb meat. Different lowercase letters indicate significant differences (*p* < 0.05).

As shown in Figure 3, the concentration of active MMPs gradually increased with prolonged storage time; however, no significant changes were observed in MMPs activity between adjacent storage times. The concentration of active MMPs in lamb meat stored for 7 days was significantly higher compared with that in lamb meat after 1-day storage.

**Figure 4:**
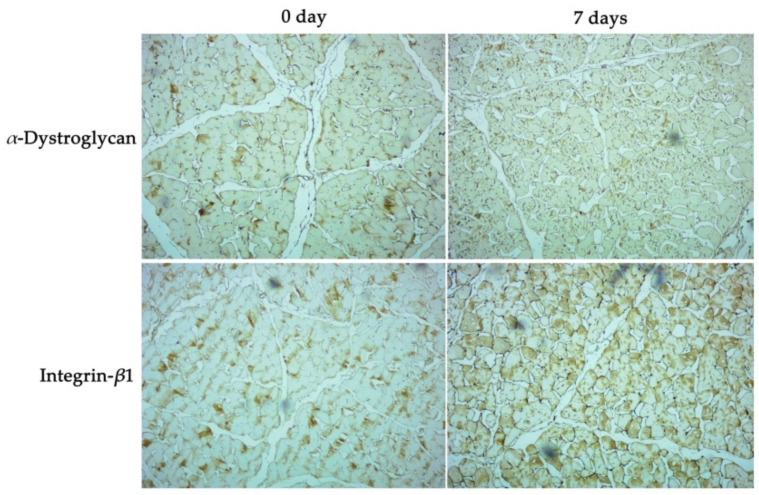
Profile of *α*-dystroglycan and integrin-*β*1 in raw lamb meat submitted to different storage times (0 and 7 days).

On day 7 postmortem, *α*-DG and integrin-*β*1 could be detected inside myofibrils, on the muscle cell surface, and the endomysium surface (Figure 4); in addition, the gap between the muscle membrane and endomysium was clearly observed, which indicated that the membrane structure was destroyed; part of the membrane was bound to the surface of the myofibril network, and the other part of the membrane was bound to the endomysium. Previous results of experiments conducted by our group with muscle at different postmortem times showed that the muscle fiber membrane of thawed muscle tissue was destroyed, and part of the membrane was separated from the myofibril network (data not shown).

## 4. Discussion

### 4.1. The Effect of Postmortem Aging and Heating on Longitudinal, Transverse and Volume Shrinkage of Lamb Meat Cubes 

During postmortem aging, connective tissue, myofibrils, and linkers between myofibrils and the endomysium are partially hydrolyzed by endogenous enzymes, such as calpain and cathepsin [32,33]. To explore whether hydrolysis is related to muscle shrinkage, the effect of aging on the structure as well as the longitudinal, transverse, and volume shrinkage of lamb meat was evaluated in the present study. The transverse and longitudinal-shrinkage ratios of heated lamb meat were significantly influenced by storage time (Table 1) (*p* < 0.05). Other studies found that the onset of longitudinal shrinkage of muscle tissue was related to storage time. With a 1-day storage, the onset of longitudinal shrinkage occurred at 70 °C [9]; with a 7-day storage, the onset of longitudinal shrinkage started at 50 °C, which occurred at a lower temperature compared with the meat samples stored for 1 day. However, in another study, it was found that both 1 d and 14 d aged beef began to shrink longitudinally at 55 °C, with an alteration of about 3% [34]. For transverse shrinkage, the shorter the storage time, the greater the shrinkage ratio when the heating temperatures were 70 °C and 90 °C [9]. Divergencies in the results mentioned above might be due to the influence of muscle type, storage time, animal breed or gender, among others. It is believed that muscle thermal shrinkage is due to the thermal denaturation of proteins such as myosin [35,36], actin [34], and collagen [13,37]. Myosin thermosensitivity is dependent upon fiber type; for example, myosin in cutaneous trunci muscle tissue was denatured at a lower temperature than in masseter muscle tissue [35]. In this article, 1 cm^3^ cubes were used to study the shrinkage of meat submitted to heat or air drying. If the meat size was bigger, the heat treatment on the surface of the meat was more intense, and the shrinkage will be different when the core temperature of the meat reaches a certain value. More research is needed to study the effect of meat size on the shrinkage ratio of meat submitted to heat or air drying.

### 4.2. Effect of Storage Aging and Heating on Porosity of Lamb Meat Cubes

The porosity of raw- or processed-lamb-meat cubes (Figure 1) was mainly related to the gap between myofibril networks and between muscle fascicles. To understand the reduction in the gap in lamb meat after heating (Figure 1), it is necessary to determine the thermal-shrinkage properties of muscle fascicles, the endomysium, and of the perimysium. Muscle cubes, muscle fragments, and myofibrils showed similar maximum cross-sectional shrinkage ratios (20–24%), but the maximum length shrinkage of the myofibrils was lower (15%) than that of the muscle cubes and fragments (25%) [34,38]. This indicated that the restriction of myofibrils by the endomysium resulted in decreased shrinkage of the myofibrils during heating at temperatures above 65 °C. At 55 °C, the myofibrils contracted by 10–15%; at 60 °C, the shrinkage ratio of the meat stored for 1 day was reduced by 0–5%, while the shrinkage ratio of the meat stored for 14 days was 15% [39]. 

The onset of transverse and longitudinal shrinkage of the isolated endomysium of bovine *sternomandibularis* muscle occurred at about 50 °C and intensified at higher temperatures. The diameter and length of the isolated endomysium could shrink to 70% and 40%, respectively, when heated at 85 °C [15]. These dimensional changes in the isolated endomysium were more remarkable than those reported in whole meat. Using various bovine muscle tissues heated at 80 °C, the length decreased by 8–30%, whereas the cross-sectional area decreased by 10–30% [9,34,40,41]; thus, when beef is heated at 80 °C, the endomysium strictly encases the muscle sarcolemma akin to a rubber band. However, in our study, when lamb meat samples were heated at 90 °C, a gap between the myofibril networks was still observed. Astruc et al. (2021) found a gap between myofibril networks in bovine rectus abdominis muscle tissue heated at 100 °C for 15 min [27]. This phenomenon might be due to the further shrinkage of myofibrils at temperatures within the range of 80–100 °C [39] or to animal breed, since the shrinkage ratio of pork meat was greater than beef [41]. In addition, the denaturation temperature of collagen in the muscle tissue of different animals differs widely, e.g., 65.3 °C in chicken and 69.2 °C in beef; moreover, the denaturation temperature in various parts of pork muscle tissue differs (approximately 60 °C) [42,43,44,45].

### 4.3. The Effect of Postmortem Aging and Heating on Connective Tissue Shrinkage of Heated Lamb Meat Cubes 

Collagen is the main component of connective tissue. However, the results of some research indicate that a higher content of thermally insoluble collagen in muscle did not necessarily result in a greater shrinkage ratio or an increase in initial shrinkage temperature [14,44]. To explain this phenomenon, Latorre, Velazquez, and Purslow (2018) used differential scanning calorimetry (DSC) to study the thermal denaturation of perimysium collagen isolated from bovine *semitendinosus* and *pectoral profundus* muscle tissues. In this study, it was found that the reversible unhelix of the collagen triple helix in *semitendinosus* connective tissue required more energy, which may be caused by the postmortem degradation of proteoglycans [13]. 

It has been shown that proteoglycan could stabilize the structure of collagen, as it has been shown that it increased intermolecular crosslinking in collagen [36,46,47]. With increased molecular crosslinking in collagen, contractile tension did not decrease at a relatively elevated temperature [48]. Further studies confirmed that the degradation of proteoglycan can modulate the heating properties of collagen [49,50]. Proteoglycans in the endomysium are gradually degraded during postmortem aging [51]. Veiseth-Kent et al. (2018) found that aggrecan in *longissimus lumborum* and *infraspinatus* muscle tissues degraded to varying degrees on days 2 and 13 postmortem aging, and the energy required for the despiralization of endomysium collagen decreased [26]. Vaskoska et al. (2021) showed that inhibition of cathepsin led to increased transverse shrinkage, reduced longitudinal shrinkage, and increased volume shrinkage of *semitendinosus* muscle fibers heated within the temperature range of 65 °C–90 °C [39]. However, in this study, it was not possible to distinguish whether changes in the shrinkage ratios in muscle fiber bundles were caused by the influence of cathepsin on muscle fibers or connective tissue, which requires further study. The endomysium is complex, and several types of proteoglycans have been described; however, the kinds of proteoglycans which are related to the shrinkage of endomysium require further study.

### 4.4. Effects of Degradation of Proteins Binding Endomysium and Myofibril Induced by Matrix Metalloproteinases (MMPs) on Gap Formation in Raw Lamb Meat 

In a previous study, the muscle fiber membrane and basement membranes were found to be close to myofibrils in muscle fibers at 0.5 h postmortem, and the integrity of the muscle fiber membrane was destroyed in the lamb meat frozen and thawed 48 h postmortem (data not shown). Other studies revealed that the basement membrane quickly detached from the muscle cell membrane, indicating that linker proteins between the two membranes were degraded at an early postmortem stage [52,53]. However, it is known that at early postmortem time, a gap was formed between myofibril and sarcolemma, and gaps are further formed between sarcolemma and endomysium with prolonged postmortem time. It has been shown that the degradation of laminin at 24 h postmortem can be observed using immunoelectron microscopy, and the cell membrane and the basement membrane were closely associated [54,55]. Furthermore, other studies found that sarcolemma and myofibrils in bovine muscle were separated at 24 h postmortem; the sarcolemma was completely degraded at 72 h postmortem [56,57]. 

The degradation of binding proteins between myofibrils and the endomysium might be important to the shrinkage of the endomysium of muscle cubes below 55 °C. The onset of shrinkage of muscle myofibril occurred at below 40 °C, whereas the onset of shrinkage of the endomysium occurred above 55 °C [15,39]. Figure 2 depicts that *β*-DG, *α*-DG, dystrophin, and integrin-*β*1 were susceptible to hydrolysis during storage; the longer the storage time, the stronger the degradation of such proteins. It has been shown that the degree of degradation of dystrophin, dystroglycan, and laminins gradually increases with prolonged postmortem time [54,55]. In addition, it has been suggested that the degradation of these proteins might be regulated, since the degradation of these proteins in PSE meat is lower than that in normal meat at 30 min, 24 h, 48 h, and 72 h postmortem [58]. Moreover, it has been demonstrated that carbohydrates are bound to laminin [59,60]; thus, the loss of carbohydrates (Figure 2) might have induced the separation of the muscle membrane and the endomysium of lamb meat. 

Initially, it has been suggested that MMP-2 and MMP-9 could degrade gelatin. However, it was further described that they could hydrolyze laminin and other linking proteins between myofibril networks and the endomysium, and additional degradation substrates of MMP-2 and MMP-9 are constantly being discovered. *β*-dystroglycan is the hydrolytic substrate of MMPs [23]. Under physiological and pathological conditions, MMP-2 could degrade *β*-DG and destroy the connection between the extracellular matrix and the cytoskeleton. Studies on *β*-DG in neurons have found that MMP-2 and MMP-9 could hydrolyze the extracellular region of *β*-DG, resulting in N-terminal shedding in the extracellular region of *β*-DG and dissociation of *α*-DG, which is originally linked to the N-terminal of *β*-DG [24]. Moreover, it has been found that integrin-*β*1 completes signal transduction with the participation of MMP-2, which affects the reorganization of the muscle cytoskeleton; thus, this process could be halted by inhibiting the activity or blocking MMP-2 [25]. Veiseth-Kent et al. showed that the activity of MMP-2 increased significantly with prolonged postmortem time [26]. However, knowledge of the effect of MMPs on connective tissue during postmortem and meat processing is still scarce. Moreover, studies exploring postmortem MMPs degradation of connective tissue and its association with sensory and technological quality of meat products are still lacking.

### 4.5. The Formation of Water Channels between Muscle Fascicles Might Play an Important Role in Shrinkage of Lamb Meat Cubes

The longer the storage time, the greater the proportion of muscle fibers that completely detach from the endomysium (Table 3), resulting in the formation of a water channel between myofibril networks, which might be induced by the degradation of proteins binding the muscle membrane and the endomysium, including dystrophin, *β*-dystroglycan, *α*-dystroglycan and integrin-*β*1 (Figure 2). Chen Tao et al. (2021) described that interfascicular pores’ size in samples with high drip loss were significantly higher than in samples with low drip loss; however, no significant difference was found in intracellular pore size between these two groups of samples, suggesting an important role of the water channel in muscle fascicles [61]. Moreover, in the present study, integrin was found distributed on the membrane and in the endomysium (Figure 4), but not on the surface of the fascium, indicating that the water channel was found between the endomysium and perimysium; this could be related to a loose link between the perimysium and muscle cells. Passerieux et al. showed the occurrence of linking areas between the perimysium and endomysium; in linking areas, the perimysium was found to be not closely associated with the endomysium, and collagen fibers in the endomysium were inserted in the muscle fibers at an angle of 60° [62,63].

### 4.6. Degradation of Protein Linking the Endomysium and Myofibril Did Not Have an Important Impact on Longitudinal, Transverse and Volume Shrinkage of 35 °C Air-Dried Lamb Meat Cubes Stored for 1 Day or Less Time

The gap between muscle bundles and between myofibril networks was not evident outside of air-dried lamb meat samples (Figure 1); this might be related to a higher degree of shrinkage of the perimysium and endomysium compared to the shrinkage degree of the muscle fibers. Thus, degradation of protein linking the connective tissue and myofibrils did not influence the transverse and volume shrinkage of lamb meat air-dried at 35 °C. Wess et al. (2000) found that the D unit length of collagen fibers shortened (from 67.2 nm to 65.1 nm) after being air-dried (21.4 ± 2.0 °C) 24 h [64]. However, research on muscle shrinkage and its mechanism during meat air drying is still lacking. Thus, it can be speculated that the shrinkage of collagen is responsible for the shrinkage of connective tissue.

## 5. Conclusions

Storage time and heating temperature influenced the shrinkage of heated lamb meat. In contrast, storage time did not influence the shrinkage of air-dried lamb meat. In addition, the microstructure of heated lamb meat was influenced by storage time. During storage, the proportion of muscle fibers that completely detached from the endomysium increased, probably due to the partial degradation of proteins or the loss of sugar chains in proteins linking connective tissue and myofibrils, including *β*-DG, integrin-*β*1, dystrophin and *α*-DG. When air-dried at 35 °C or water-bath-heated at 90 °C, the degradation of these proteins did not influence the shrinkage of lamb meat since the distance between myofibril networks decreased, which could be due to the higher shrinkage ratio of the endomysium compared to that of myofibers. The effect of these linking proteins on the shrinkage of heated lamb meat (raw meat stored 1 day or less time) requires further elucidation.

## Figures and Tables

**Table 1:** Effect of storage time (1, 5 and 7 days) and water bath heating temperature (50 °C, 70 °C, and 90 °C) on longitudinal, transverse and volume shrinkage of lamb meat samples.

	Storage Time	Heating Temperature			*p* Value		
		50 °C	70 °C	90 °C	Storage Time	Heating Temperature	Storage Time × Heating Temperature
Longitudinal-shrinkage ratio (%)	1 day	−0.31 ± 3.60 ^Ac^	6.75 ± 1.68 ^Bb^	25.95 ± 4.52 ^Aa^	0.006	<0.001	0.600
	5 days	0.55 ± 4.33 ^Ac^	13.71 ± 4.16 ^Ab^	30.27 ± 2.32 ^Aa^			
	7 days	4.30 ± 2.42 ^Ac^	13.29 ± 3.14 ^Ab^	31.38 ± 0.83 ^Aa^			
Transverse-shrinkage ratio (%)	1 day	8.69 ± 5.30 ^Ab^	24.59 ± 4.49 ^Aa^	28.20 ± 6.11 ^Aa^	0.001	<0.001	0.892
	5 days	4.80 ± 2.28 ^Ab^	22.88 ± 4.43 ^ABa^	23.78 ± 3.20 ^ABa^			
	7 days	2.13 ± 2.89 ^Ab^	16.50 ± 0.56 ^Ba^	18.56 ± 1.78 ^Ba^			
Volume shrinkage ratio (%)	1 day	6.47 ± 4.49 ^c^	39.60 ± 5.17 ^b^	53.46 ± 0.43 ^a^	0.967	<0.001	0.427
	5 days	3.05 ± 17.40 ^c^	41.38 ± 8.64 ^b^	53.95 ± 4.97 ^a^			
	7 days	10.36 ± 5.40 ^c^	38.08 ± 3.03 ^b^	52.40 ± 3.75 ^a^			

All data are presented as means ± standard deviation. Lamb samples were submitted consecutively to the three heating treatments (50 °C, 70 °C and 90 °C); heating holding time was 20 min once the desired temperature was reached. Means of longitudinal, transverse or volume shrinkage ratio of samples heated at the same temperature with different capital letters indicate significant storage time differences (*p* < 0.05). Means of longitudinal, transverse or volume shrinkage ratio of samples stored for the same time with different lowercase letters indicate significant temperature differences (*p* < 0.05).

**Table 2:** Effect of storage time (1, 5, and 7 days) on longitudinal, transverse and volume shrinkage of air-dried lamb meat.

	1 Day	5 Days	7 Days	*p* Value
Longitudinal-shrinkage ratio (%)	94.70 ± 3.56	90.17 ± 6.84	88.77 ± 4.25	0.387
Transverse-shrinkage ratio (%)	78.73 ± 9.89	77.88 ± 2.55	85.10 ± 4.76	0.390
Volume shrinkage (%)	54.51 ± 5.79	58.93 ± 6.91	59.97 ± 5.83	0.553

All data are presented as means ± standard deviation. Air-drying temperature was 35 °C until the moisture content of air-dried lamb samples reached 50%.

**Table 3:** Effect of storage time (1, 5, and 7 days) on the proportion of muscle fibers in lamb meat completely detached from endomysium (%).

	1 Day	5 Days	7 Days	*p* Value
Raw lamb muscle	5.31 ± 3.22 ^c^	9.23 ± 3.30 ^b^	13.67 ± 7.25 ^a^	0.012

All data are presented as means ± standard deviation. Different lowercase letters within each row indicate significant differences (*p* < 0.05).

**Table 4:** Effect of storage time (1, 5, and 7 day), water bath heating (consecutively to the three heating treatments: first 50 °C, heated for 20 min; then 70 °C, heated for 20 min; finally 90 °C, for heated 20 min) or air drying (35 °C drying until moisture content reached 50%) on lamb muscle structure.

Parameter	Treatment (Heating or Air Drying)	Storage Time			*p* Value		
		1 Day	5 Days	7 Days	Storage Time	Treatment	Storage Time× Treatment
Distance between muscle bundles (μm)	Raw meat	4.88 ± 1.91 ^Aa^	4.15 ± 1.23 ^Ab^	3.70 ± 2.22 ^Ac^	<0.001	0.002	0.424
	Outside heated meat	3.71 ± 1.23 ^Ca^	3.49 ± 1.88 ^Ba^	2.59 ± 1.00 ^Cb^			
	Inside heated meat	4.35 ± 1.64 ^Ba^	3.51 ± 1.48 ^Bb^	2.99 ± 1.38 ^Bc^			
	Raw meat	4.88 ± 1.91 ^Aa^	4.15 ± 1.23 ^Bb^	3.70 ± 2.22 ^Bb^	<0.001	<0.001	0.074
	Outside dried meat	2.29 ± 1.09 ^Cb^	2.54 ± 1.19 ^Cb^	3.43 ± 2.95 ^Ba^			
	Inside dried meat	4.00 ± 2.61 ^Bc^	4.65 ± 2.56 ^Ab^	6.34 ± 5.84 ^Aa^			
Lamb meat Porosity (%)	Raw meat	49.07 ± 4.91 ^a^	45.19 ± 9.30 ^b^	37.16 ± 9.56 ^c^	<0.001	<0.001	0.005
	Outside heated meat	19.72 ± 5.85 ^g^	28.21 ± 21.14 ^e^	22.58 ± 5.45 ^f^			
	Inside heated meat	29.38 ± 3.60 ^e^	33.54 ± 15.23 ^d^	27.86 ± 3.33 ^ef^			
	Raw meat	49.07 ± 4.91 ^a^	45.19 ± 9.30 ^b^	37.16 ± 9.56 ^c^	0.058	<0.001	0.008
	Outside dried meat	17.04 ± 8.86 ^d^	20.28 ± 9.66 ^d^	18.62 ± 9.74 ^d^			
	Inside dried meat	36.92 ± 13.92 ^c^	45.05 ± 8.55 ^b^	38.33 ± 12.64 ^c^			
Distance between myofibril networks (μm)	Raw meat	2.23 ± 0.64 ^Aa^	2.15 ± 0.64 ^Aa^	1.75 ± 1.02 ^Ab^	0.001	<0.001	0.940
	Outside heated meat	1.41 ± 0.70 ^Ba^	1.26 ± 0.56 ^Bb^	0.88 ± 0.49 ^Bc^			
	Inside heated meat	1.49 ± 0.71 ^Ba^	1.17 ± 0.51 ^Bb^	0.92 ± 0.41 ^Bc^			
	Raw meat	2.23 ± 0.64 ^A^	2.15 ± 0.64 ^A^	1.75 ± 1.02 ^A^	0.104	<0.001	0.474
	Outside dried meat	0.88 ± 0.31 ^C^	0.84 ± 0.32 ^C^	0.78 ± 0.47 ^C^			
	Inside dried meat	1.45 ± 0.72 ^B^	1.73 ± 0.58 ^B^	1.39 ± 0.83 ^B^			
Area of myofibril networks (μm^2^)	Raw meat	65.03 ± 17.98 ^Ab^	64.14 ± 21.57 ^Ab^	86.72 ± 32.21 ^Aa^	0.019	<0.001	0.101
	Outside heated meat	53.45 ± 16.52 ^Ba^	48.31 ± 16.91 ^Cb^	53.01 ± 16.59 ^Ca^			
	Inside heated meat	52.92 ± 14.07 ^Bb^	57.04 ± 19.19 ^Bab^	58.88 ± 14.31 ^Ba^			
	Raw meat	65.03 ± 17.98 ^d^	64.14 ± 21.57 ^d^	86.72 ± 32.21 ^a^	0.179	<0.001	<0.001
	Outside dried meat	61.89 ± 15.74 ^de^	59.31 ± 19.04 ^e^	45.61 ± 13.13 ^f^			
	Inside dried meat	74.81 ± 15.17 ^c^	79.57 ± 47.36 ^b^	65.69 ± 19.02 ^d^			
Endomysium circumference of muscle (μm)	Raw meat	31.81 ± 4.23 ^Aa^	32.18 ± 4.43 ^Aa^	31.41 ± 4.90 ^Aa^	<0.001	<0.001	0.449
	Outside heated meat	27.32 ± 2.86 ^Bb^	29.61 ± 3.22 ^Ba^	29.27 ± 4.90 ^Ca^			
	Inside heated meat	27.49 ± 3.93 ^Bb^	30.41 ± 4.87 ^Ba^	30.15 ± 5.53 ^Ba^			
	Raw meat	31.81 ± 4.23 ^ab^	32.18 ± 4.43 ^ab^	31.41 ± 4.90 ^b^	<0.001	<0.001	<0.001
	Outside dried meat	25.71 ± 2.97 ^e^	26.82 ± 4.78 ^d^	24.87 ± 3.34 ^e^			
	Inside dried meat	29.31 ± 2.98 ^c^	32.84 ± 4.56 ^a^	29.02 ± 3.97 ^c^			

All data are presented as mean ± standard deviation. When the 2-way interaction between storage time and treatment was not significant, means of the trait stored the same time with different capital letters indicating significant treatment differences (*p* < 0.05); means of the trait treated the same with different lowercase letters indicate significant storage time differences (*p* < 0.05). When the 2-way interaction between storage time and treatment was significant, means of every trait of heated or air-dried samples with different lowercase letters indicate significant different (*p* < 0.05).

## Data Availability

The data presented in this study are available within the article.
